# Screen-Printed Fabrication of PEDOT:PSS/Silver Nanowire Composite Films for Transparent Heaters

**DOI:** 10.3390/ma10030220

**Published:** 2017-02-23

**Authors:** Xin He, Ruihui He, Qiuming Lan, Weijie Wu, Feng Duan, Jundong Xiao, Mei Zhang, Qingguang Zeng, Jianhao Wu, Junyan Liu

**Affiliations:** School of Applied Physics and Materials, Wuyi University, Jiangmen 529020, Guangdong, China; ruihuihe@yeah.net (R.H.); wyuqiuminglan@yeah.net (Q.L.); wjwu2016@126.com (W.W.); duanfeng0922@163.com (F.D.); jundongxiao99@163.com (J.X.); zmjenny@163.com (M.Z.); zengqg1979@126.com (Q.Z.); jhwuwyu@126.com (J.W.); liujunyanwyu@126.com (J.L.)

**Keywords:** screen printing, conductive film, transparent heater

## Abstract

A transparent and flexible film heater was fabricated; based on a hybrid structure of poly(3,4-ethylenedioxythiophene) poly(styrenesulfonate) (PEDOT:PSS) and silver nanowires (Ag NWs) using a screen printing; which is a scalable production technology. The resulting film integrates the advantages of the two conductive materials; easy film-forming and strong adhesion to the substrate of the polymer PEDOT:PSS; and high conductivity of the Ag NWs. The fabricated composite films with different NW densities exhibited the transmittance within the range from 82.3% to 74.1% at 550 nm. By applying 40 V potential on the films; a stable temperature from 49 °C to 99 °C was generated within 30 s to 50 s. However; the surface temperature of the pristine PEDOT:PSS film did not increase compared to the room temperature. The composite film with the transmittance of 74.1% could be heated to the temperatures from 41 °C to 99 °C at the driven voltages from 15 V to 40 V; indicating that the film heater exhibited uniform heating and rapid thermal response. Therefore; the PEDOT:PSS/Ag NW composite film is a promising candidate for the application of the transparent and large-scale film heaters.

## 1. Introduction

Transparent film heaters are widely used in defogging vehicle windows, heating outdoor displays, or thermal therapies [1,2,3,4,5,6]. They are known for converting electric energy into heat energy, and maintaining transparency. The temperature of the heater can be accurately controlled by changing the input voltages. The most common transparent-heater is represented by indium tin oxide (ITO) [7]. However, the limited reserves and brittleness limit its further application in the next-generation devices. Additionally, ITO exhibits a slow temperature response because of its intrinsic nature.

Several emerging nanomaterials are developed as transparent film heaters to substitute for ITO, such as conductive polymers [8], carbon nanotubes [9,10], grapheme [1,11,12,13], metal meshes [14] and nanowires [2,4,15,16,17,18]. However, there are several obstacles for these materials to overcome before wide application in the film heater. For example, the conductive polymer PEDOT:PSS can be easily fabricated as a uniform film, but its conductivity usually requires further improvement by chemical modifications to achieve a high-performance heater [8,19,20]. Among these materials, the film heater using the metal nanowires has shown excellent optoelectronic and heating performance. However, the poor adhesion of metal network to the substrate limits its wide applications. Researchers have used many strategies to address this issue. For instance, Ghosh et al. prepared a transparent film by means of comprising Ag nanowires (NWs) in an ultrathin polyimide (PI) foil, exhibiting high transmittance (>90%), strong adhesion, and low surface roughness of 2.4 nm. The Ag NW-PI film with a sheet resistance of 15 Ω/sq can generate a high temperature exceeding 100 °Cat 17 V within 20 s [21].

To represent the advantages of various emerging-materials, two conductive structures are usually hybridized to fabricate the film heater with high performance. For example, Choi et al. introduced an Ag-grid/graphene hybrid structure using the hot-pressing transfer and electrohydrodynamic jet printing, exhibiting the superior electrical properties and heating performance [22]. Sung and Kim et al. reported a flexible Ag NW-polymer composite electrode with the transmittance of 84.3% and sheet resistance of 10.76 Ω/sq via a continuous two-step spray-coating method. The conductivity of PEDOT:PSS was improved by the use of dimethyl sulfoxide [20]. In general, the preparation of the transparent film heaters in many publications requires spin-coating [21], vacuum filtration [23], transfer process [5,17] or repeated coating [24]. Additionally, a chemical or heating treatment is normally needed for improving the film performance [8,11,20]. Thus, the large-scale production of the film heater is greatly restricted.

The screen printing is an environment-friendly and cost-effective method to fabricate uniform films. It is one of the scalable production technologies [25,26,27]. In this study, a composite film has combined PEDOT:PSS with Ag NWs using a screen-printed fabrication to gain a uniform film with good conductivity. The whole process was carried out at the room temperature without any annealing or chemical treatment. The effects of the mesh number and Ag NW concentration on the optoelectronic properties of the printed films were demonstrated. The conductivity and adhesion of the films were simultaneously improved as compared in the case of using one printing material. The PEDOT:PSS/Ag NW composite film exhibited uniform heating and rapid thermal response. The film with the transmittance of 74.1% at 550 nm could generate a steady temperature of 41 °C at 15 V within 35 s. This reveals the promising application in the large-area, transparent and flexible film heater.

## 2. Experimental Section

### 2.1. Synthesis of Long Ag NWs

Long Ag NWs were synthesized according to a hydrothermal method [28]. Typically, 2 mmol of glucose, 1.5 mmol of silver nitrate, and 0.3 mmol ferric sulfate were, respectively, dissolved withdeionized (DI) water (the volume ratio of reactantsis 2:2:1) in a beaker at room temperature. Then, the reactants were mixed together and magnetically stirred for several minutes to yield a light-yellow solution. Following this, 4.5 g polyvinylpyrrolidone (PVP, K30) was introduced into it. The mixture was consistently stirred until PVP totally dissolved, and then transferred into a Teflon autoclave of 100 mL capacity, which was sealed and heated at 180 °C for 6 h. After the hydrothermal treatment, the gray-green precipitate was obtained. Then, the precipitate was washed with dilute nitric acid solution for several times in order to get rid of the oxide layer on the NW surfaces. The excess nitric acid was removed by centrifugations using ethanol. The long Ag NWs could be collected by repeated suction filtration. Finally, the product was kept in ethanol to obtain a dispersion of long Ag NWs. The diameter of the NWs is approximately 100 nm, and the length is up to hundreds of micrometers.

### 2.2. Fabrication of PEDOT:PSS/Ag NW Composite Films

PEDOT:PSS (Orgacon, EL-P3145, Agfa, Mortsel, Belgium)/Ag NW composite films were fabricated by the screen-printed technology on the polyethylene terephthalate (PET) substrate. Before printing, the substrates were washed by ethanol and deionized water respectively with assistance of ultrasonic treatment for 15 min to remove the dust, grease and other impurities on the substrate surface. After that, 4, 6, 8, and 12 mg of dried Ag NWs were respectively added into 1 mL PEDOT:PSS suspension, and then the mixtures were stirred intensively to obtain homogeneous PEDOT:PSS/Ag NW inks.

**Figure 1:**
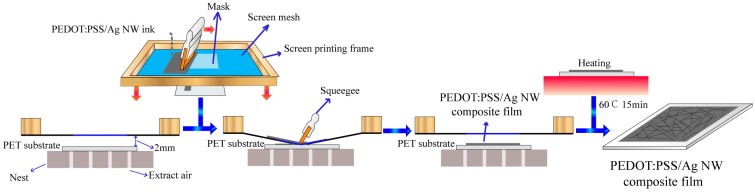
Schematic illustration of the screen-printed procedure of PEDOT:PSS/Ag NW composite films. PEDOT:PSS: poly(3,4-ethylenedioxythiophene) poly(styrenesulfonate); NW: nanowire.

Figure 1 shows a schematic illustration of fabrication procedure of the PEDOT:PSS/Ag NW composite film. Firstly, the PET substrate was adsorbed on the nest by mechanical pump, and then the cleaned printing mesh was adjusted to maintain an appropriate distance of 2 mm with the substrate. Following this, a PEDOT:PSS/Ag NW ink was dropped onto the mask, and the squeegee quickly pushed toward another side. The printed films were finally heated at 60 °C for 15 min.

### 2.3. Characterization

The surface morphologies of the printed films were observed using a field emission scanning electron microscope (FESEM, Nova NanoSEM 430, FEI, Hillsboro, OR, USA). The nanostructures of the films were characterized using an X-ray diffractometer (XRD) (X’pert Pro MFD, Panalytical, Almelo, The Netherlands) with a Cu Kα radiation (λ = 0.154178 nm). The transmittances were collected by a UV-VIS spectrophotometer (UV2550, Shimadzu, Kyoto, Japan). The resistances of the films were recorded by a multimeter (MS8229, MASTECH, Moon, PA, USA) and the sheet resistances of the films were characterized by a four-probe system (SZ-82, Suzhou Telecommunication, Suzhou, China). The heating performances of the films were measured using a two-terminal side contact configuration. The applied DC voltage was supplied by a power supply (IT6720, ITECH, San Luis Obispo, CA, USA) to the heater through two copper conductive tapes pasted at the film edges. The temperatures and heat distributions of the transparent films were measured using an IR thermal imager (Ti32, Fluke, Washington, DC, USA).

## 3. Results and Discussion

**Figure 2:**
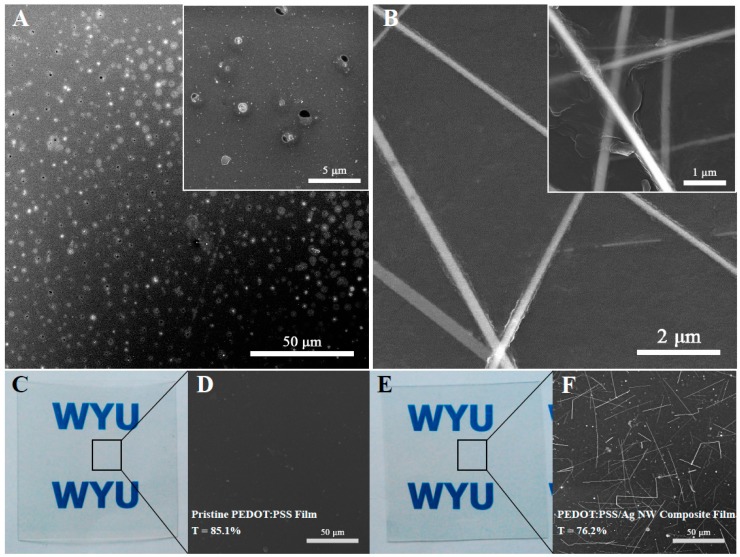
SEM images of PEDOT:PSS film using a 420 mesh (**A**) and PEDOT:PSS/Ag NW film using a 350 mesh (**B**); the insets were magnified images. Photographs (**C**,**E**) and SEM images (**D**,**F**) of pristine PEDOT:PSS and PEDOT:PSS/Ag NW film printed using a 350 mesh, and the added Ag NW concentration was 8 mg/mL.

The mesh number was one of key factors to decide the film properties in the screen-printed process. In the experiment, the uniform films could be fabricated as the mesh number was in the range of 200–350. The mesh size was, respectively 75, 60, 50, and 43 μm, corresponding to the mesh number 200, 250, 300, and 350. As shown, the mesh size was decreased with the increase of the mesh number. When the mesh number was below 200, the tension force of conductive ink was not enough to form a stable ink column due to a large mesh size. The ink would cross the mesh because of the gravity before printing, which could lead to the fabrication of a heterogeneous film. In case the mesh number exceeded 350, the mesh size would be too small to make an ink that would fully cover the PET substrate, and the uniformity of the composite film would be damaged. Figure 2A presented a typical SEM image of pristine PEDOT:PSS film printed using a 420 mesh, and many small bubbles and holes were observed on the surface, generating an heterogeneous film. For comparison, Figure 2B displayed a SEM image of the PEDOT:PSS/Ag NW composite film printed with a 350 mesh. No obvious bubble or hole could be found on the film surface. The inset was a magnified SEM image of the NW connections, suggesting that the NW surfaces had been covered by polymer PEDOT:PSS. The Ag NW network could provide more electron pathways, and greatly enhanced the conductivity of the composite film. Meanwhile, the coverage of PEDOT:PSS on the Ag NWs could isolate the NWs from the air, preventing the oxidation of metallic Ag NWs and enhancing the adhesion to the PET substrate.

The 350 mesh was chosen to fabricate the films with high transparency and good uniformity. Figure 2C–F showed the photographs and typical SEM images of the pristine PEDOT:PSS and PEDOT:PSS/Ag NW film printed by a 350 mesh, respectively. The addition of Ag NWs in the inks was 8 mg/mL. The transmittance of pristine PEDOT:PSS film was decreased from 85.1% to 76.2% when 8 mg/mL Ag NWs were mixed with PEDOT:PSS ink. Nevertheless, the composite film mentioned above showed distinct visibility and uniform distribution of the NWs on the substrate (Figure 2B,F).

**Figure 3:**
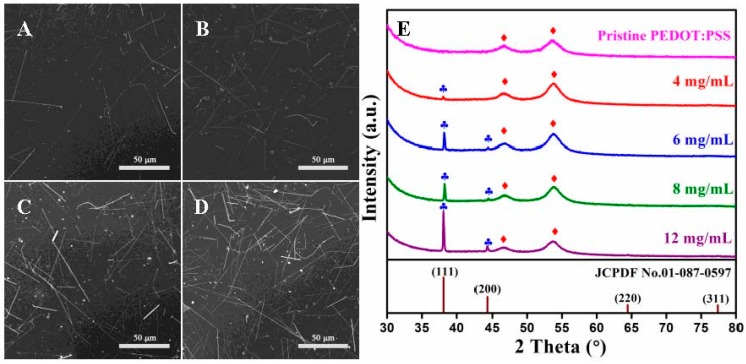
(**A**–**D**) SEM images and (**E**) XRD patterns of PEDOT:PSS/Ag NW composite films printed using the 350 mesh, and the Ag NW concentration was respectively 4, 6, 8 and 12 mg/mL in the ink.

To further analyze the relationship between the concentration of Ag NWs and the photoelectric properties of the composite film, the PEDOT:PSS/Ag NW composite films were fabricated with various additions of Ag NWs into the PEDOT:PSS inks. Figure 3A–D shows the SEM images of the composite films printed using the 350 mesh with the Ag NW concentration of 4, 6, 8, and 12 mg/mL, respectively. As the length of the Ag NWs was larger than the mesh size, thus, several NWs got curved and broken due to the extrusion in the printing process. When the Ag NW concentration increased, this made the crossed network in the film more intensive, which led to the increase in the conductivity while the transmittance declined for the composite films. Additionally, the NW concentration exceeded 12 mg/mL, and the NWs were hardly dispersed in the ink.

Figure 3E demonstrates the XRD patterns of the pristine PEDOT:PSS and PEDOT:PSS/Ag NW films with Ag NW concentration ranging from 4 mg/mL to 12 mg/mL, and the standard card of metallic silver was provided for reference. The two wide diffraction peaks in all films labeled by red diamonds were centered at 46.6° and 53.5°, which could be assigned to the presence of PEDOT:PSS. A weak diffraction peak at 38.1° was observed from the composite film with NW concentration of 4 mg/mL. As the addition of Ag NWs increased, the intensity of the peak at 38.1° was gradually enhanced, and a new peak at 44.4° appeared. The two diffraction peaks marked with blue clubs matched well with the (111) and (200) reflection of the face centered cubic structure of metallic silver (JCPDF No. 01-087-0597), respectively. Therefore, the content of the silver in the composite film was increased with the density of the Ag NWs in the ink. Furthermore, no other peaks could be observed, indicating the absence of other impurities in the composite films. 

**Figure 4:**
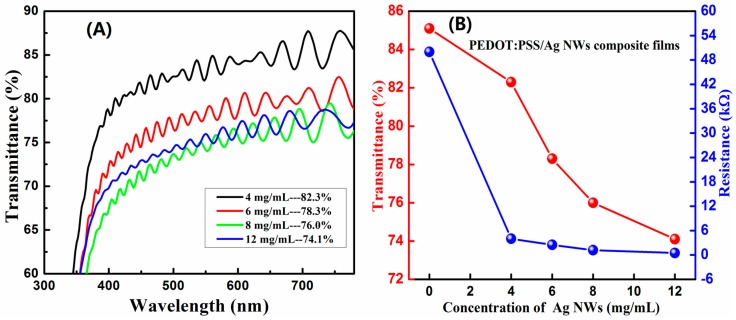
(**A**) Optical transmittance spectra and (**B**) the variation of the transmittance at 550 nm and resistance of PEDOT:PSS/Ag NW composite films printed using the 350 mesh with the concentration of Ag NWs ranging from 4 to 12 mg/mL.

Figure 4A presents the optical transmittance spectra of PEDOT:PSS/Ag NW composite films printed using the 350 mesh with Ag NW concentration ranging from 4 mg/mL to 12 mg/mL. Compared to the pristine PEDOT:PSS film with transmittance of 85% and resistance of 50 kΩ, the transmittance of the composite films was decreased from 82.3% to 74.1% at 550 nm, and the resistance was reduced from 4 to 0.5 kΩ. The areas of the printed films were approximately 50 mm × 50 mm. The changes in the transmittance and resistance of the composite films with the variation of Ag NW concentration are displayed in Figure 4B. It also shows that the transmittance of the film was slightly decreased and the conductivity of the film was greatly increased by the introduction of the conducting Ag NWs. The increasing conductivity of the composite films was caused by the percolation network of Ag NWs, which provides effective electron pathways. In the characterization process, it was found that the sheet resistance of the pristine PEDOT:PSS film was approximately 380 Ω/sq, exhibiting uniform distribution on the film surface. However, the conductivity of the composite films with a high NW density was difficult to be evaluated using the sheet resistance, which varied from several to hundreds of Ohms per square at the different locations on the film surface. We assume that the uneven distribution of the sheet resistance was brought by the Ag NWs that might randomly be exposed to air/embedded into polymer during the screen-printed process. The surface where Ag NWs were naked had low sheet resistance, while the surface where Ag NWs were embedded exhibited high sheet resistance.

**Figure 5:**
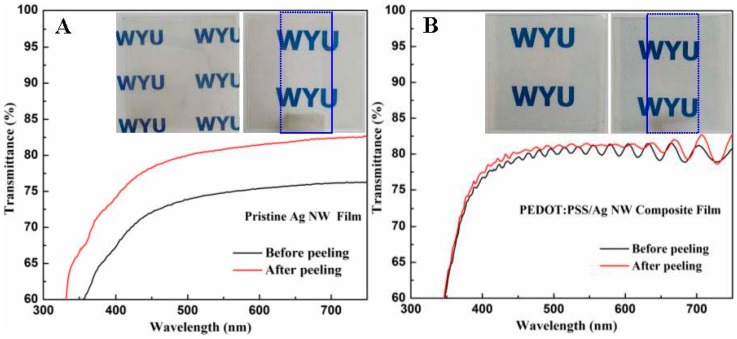
Optical transmittance spectra of the pristine Ag NW film (**A**) and the PEDOT:PSS/Ag NW composite film (**B**) before and after peeling of the tape, the insets show photographs of the films, and the blue dotted rectangles correspond to the peeling areas.

The adhesions of the films to the PET substrate were tested using 3M Scotch tape. For comparison, a pristine Ag NW film was fabricated by rod coating. Figure 5A,B display optical transmittance spectra of the Ag NW film and PEDOT:PSS composite film before and after peeling of the tape. The transmittance at 550 nm was increased from 75% to 81% for the Ag NW film. However, the transmittance of the composite film was maintained at 79%. The insets show photographs of the films before and after peeling, revealing that the Ag NW film on PET substrate was easy to peel off, and the composite film was hardly detached from the surface by the tape. The improved adhesion of the Ag NW network could be mainly attributed to the compact contact between Ag NWs and PEDOT:PSS and good adhesion of the polymer to the substrate.

**Figure 6:**
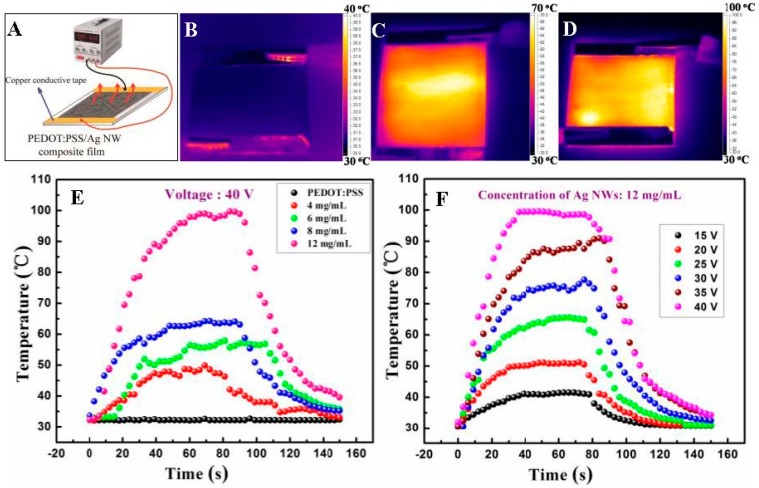
(**A**) Schematic illustration of a film heater based on the PEDOT:PSS/Ag NW composite film; infrared thermal images of the pristine (**B**) and composite film with NW concentration of 8 mg/mL (**C**); 12 mg/mL (**D**); the applied voltage was 40 V; (**E**) evolution of the generated temperature of the pristine and composite films with the different Ag NW concentrations ranging from 4 mg/mL to 12 mg/mL, the driving voltage was 40 V; and (**F**) evolution of the generated temperature of the composite film with the Ag NW concentration of 12 mg/mL at various voltages changing from 15 V to 40 V.

We investigated the heating performances of the composite films, which were measured using a two-terminal side contact configuration. A schematic illustration of the film heater is presented in Figure 6A. Figure 6B–D displays the typical infrared thermal images of the pristine PEDOT:PSS and PEDOT:PSS/Ag NW films with NW concentration of 8 and 12 mg/mL at 40 V, respectively. To improve the image contrast of the heating films, the range of the temperature bars were set as 30–40 °C, 30–70 °C and 30–100 °C. The saturable temperatures of the films were measured to be 32, 65, and 99 °C, respectively. 

The surface temperature of the pristine PEDOT:PSS film was not elevated compared to the room temperature even at 40 V. However, once Ag NWs were added into the ink, the printed composite films could generate higher temperature and more heat, which was ascribed to the improvement of the film conductivity.

Figure 6E shows the temperature evolution of the pristine and composite films with the Ag NW addition of 4, 6, 8, and 12 mg/mL in the ink, respectively. All films were driven at 40 V for 150 s. By passing the current through the films, they could be heated from room temperature to a steady temperature of 32, 49, 59, 65, and 99 °C within 30 s to 50 s, respectively. The temperature was elevated as the Ag NW density in the film increased, demonstrating that more electron pathways was beneficial for the thermal generation. Figure 6F presents the temperature profiles of the PEDOT:PSS/Ag NW composite film with the NW concentration of 12 mg/mL, which was plotted with the respect to the different voltages from 15 V to 40 V. When the potential was set as 15, 20, 25, 30, 35, and 40 V, the composite film reached the temperature of 41, 51, 66, 78, 91, and 99 °C, respectively, indicating that the generated temperature of the film raised as the applied voltage increased.

**Figure 7:**
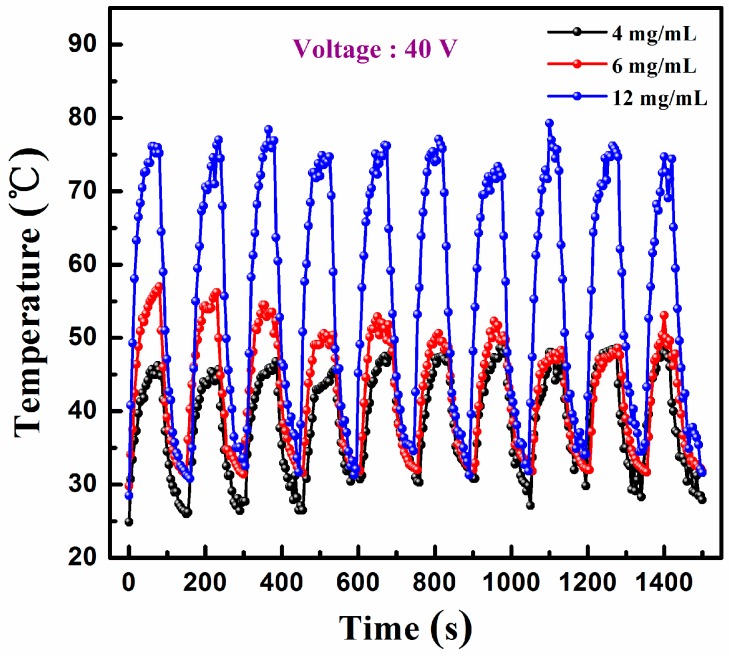
On/off responses of the film heaters with the Ag NW addition of 4, 6, and 12 mg/mL at 40 V after four-month preservation.

Figure 7 shows the on/off response of the film heaters with the Ag NW addition of 4, 6, and 12 mg/mL; the samples, which have been preserved for four months were characterized under a driving potential of 40 V. The heating and cooling time in each cycle was 80 and 70 s, respectively. The heating temperature was in the range of 47–49 °C, 49–57 °C, and 76–79 °C for the films with the Ag NW addition of 4, 6 and 12 mg/mL, respectively. The cycling curve showed relatively stable temperature recoverability of the heater based on the PEDOT:PSS/Ag NW composite films. Compared to the fresh-fabricated films with the saturable temperature of 49, 59, and 99 °C at 40 V, the films with the Ag NW addition of 4 and 6 mg/mL exhibited a stable temperature, and the film with the Ag NW addition of 12 mg/mL showed a decrease in temperature after four months. We suppose that the film with a large Ag NW density was more easily oxidized during the process of preservation, leading to the decrease of the heating temperature. The result agreed with the previous discussion. In the case of the Ag NWs with a high concentration, the composite film exhibited inhomogeneous sheet resistance because the NWs were randomly exposed to air or embedded into polymer during the printing process. After a period of preservation, the exposed NWs were prone to be oxidized, resulting in the decrease of the saturable temperature of the composite film.

The discussion above suggests that the pristine PEDOT:PSS film showed no obvious temperature increase even at 40 V. Its poor heating performance was attributed to its high resistance of the commercial PEDOT:PSS in the study, which could be improved by introducing a small amount of Ag NWs. The composite films could rapidly be heated within 30 s to 50 s. The composite films could combine the advantages of the Ag NWs with good conductivity and the polymer PEDOT:PSS with easy film-forming and strong adhesion. By adding the Ag NWs with a low density into the ink, the heating stability of the composite film could be maintained at least for four months due to the protection of PEDOT:PSS from the air. Therefore, we propose that the film heater with large area could be fabricated by scalable screen printing, revealing a bright application prospect in a large-area heating production, such as defogging in vehicles, heating in outdoor displays, or thermal therapies.

## 4. Conclusions

In this paper, a hybrid structure of PEDOT:PSS and Ag NWs was experimentally fabricated using a scalable screen-printed technology, resulting in a transparent and conductive film. The uniformity and optoelectronic properties of the fabricated films were influenced by the mesh size in the fabrication process. A uniform film could be printed with a mesh number ranging from 200 to 350. The addition of the Ag NWs into PEDOT:PSS ink brought a slight decrease in the film transmittance and significantly increased the film conductivity. Ascribed to the improvement of the optoelectronic properties, the composite films exhibited fast response and uniform temperature distribution. This work is expected to be helpful for the development of the transparent conducting films for the fabrication of large-scale, uniform film heaters.
